# Effects of task-based mirror therapy on upper limb motor function in hemiplegia: study protocol for a randomized controlled clinical trial

**DOI:** 10.1186/s13063-024-08081-1

**Published:** 2024-04-11

**Authors:** Hongzhen Liu, Yangjie Xu, Wei Jiang, Fangchao Hu, Yi Zhou, Lu Pan, Feng Zhou, Ying Yin, Botao Tan

**Affiliations:** 1 Department of Rehabilitation Medicine, The Second Affiliated Hospital of Chongqing Medical University, 74 Lin Jiang Road, Chongqing, 40010 China; 2 Department of Mechanical Engineering, Chongqing University of Technology, No. 69 Hongguang Avenue, Chongqing, 400054 China

**Keywords:** Task-based mirror therapy, Stroke, Hand function rehabilitation, Diffusion tensor imaging

## Abstract

**Background and purpose:**

Research to date has lacked definitive evidence to determine whether mirror therapy promotes the recovery of upper extremity function after stroke. Considering that previous studies did not stratify patients based on structural retention, this may be one of the reasons for the negative results obtained in many trials. The goal evaluates the efficacy of TBMT (utilizing an innovatively designed mirror) versus standard occupational therapy for stroke patient’s upper limb functionality.

**Methods and analysis:**

This single-center randomized controlled trial will involve 50 patients with stroke. All patients will be randomly assigned to either the task-based mirror therapy or the control group. The interventions will be performed 5 days per week for 4 weeks. The primary outcomes will be the mean change in scores on both the FMA-UE and modified Barthel Index (MBI) from baseline to 4 weeks intervention and at 12 weeks follow-up between the two groups and within groups. The other outcomes will include the Action Research Arm Test (ARAT), the Nine Hole Peg Test (9HPT), the Functional Independence Measure, and MRI.

**Discussion:**

This trial will not only to establish that task-based mirror therapy (TBMT) could improve the recovery of hand function after stroke but also to explore the underlying mechanisms. We expect that this finding will clarify the brain activation and brain network mechanisms underlying the improvement of hand function with task-oriented mirror therapy and lead to new ideas for stroke hand function rehabilitation.

**Trial registration:**

URL: https://www.chictr.org.cn; Unique identifier: ChiCTR2300068855. Registered on March 1, 2023

**Supplementary Information:**

The online version contains supplementary material available at 10.1186/s13063-024-08081-1.

## Introduction

The severe consequences of stroke often result in residual motor, sensory, cognitive, urinary, and fecal dysfunction, making it challenging for survivors to reintegrate into their families and society [1, 2]. Recent research indicates that approximately 85% of stroke patients experience hemiparesis in their upper or lower extremities, while 55 to 75% of stroke survivors face limited upper extremity function [3]. Upper extremity function accounts for 60% of total body function, and hand function alone comprises 90% of upper extremity function. Therefore, effective hand rehabilitation is essential to successfully reintegrate patients into their families and enhance their independence [4].

**Fig. 1:**
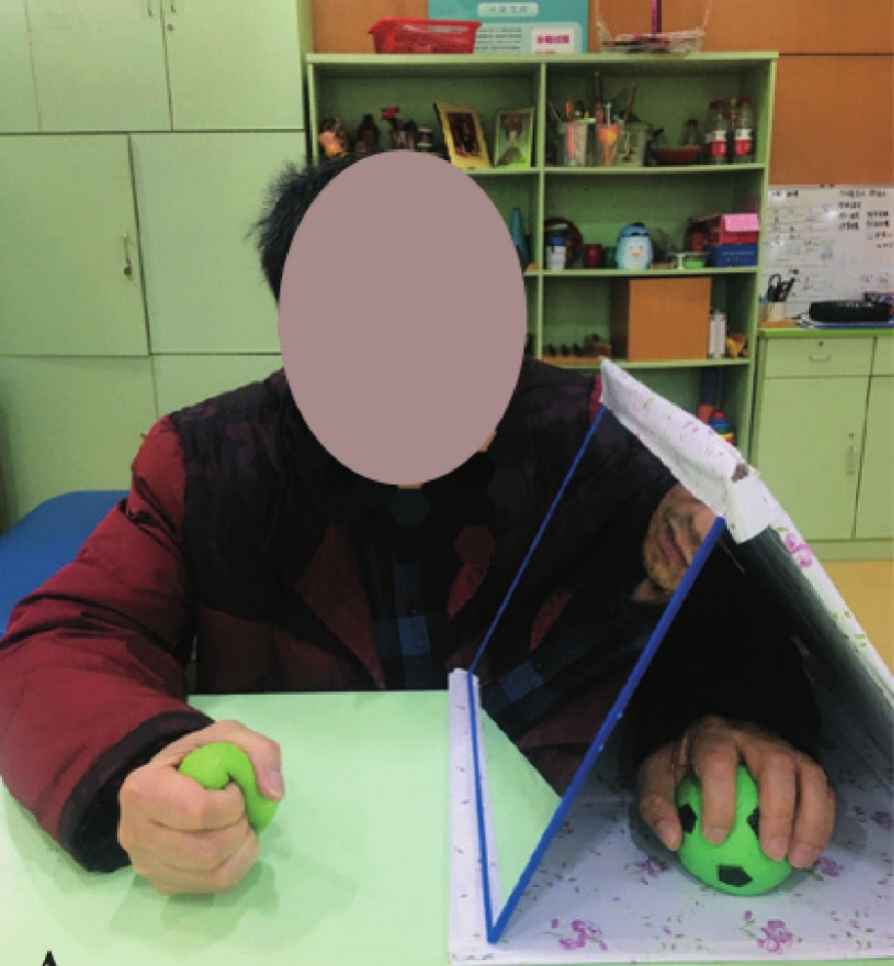
Conventional mirror therapy

Although rehabilitation training is the primary treatment method for stroke patients, the current results of rehabilitation for poststroke hand dysfunction are not satisfactory [1]. There is an urgent need to develop more effective and inexpensive rehabilitation techniques for hand function. Mirror therapy (MT) has emerged as a promising new rehabilitation intervention in recent years [5, 6]. MT typically involves placing a flat mirror on the unaffected side of the body, allowing the patient to “embody” their paralyzed limb through the optical illusion created by the mirror. This technique effectively “tricks” the brain into perceiving movement in the affected limb, potentially aiding in the recovery of motor function. MT has shown particular efficacy in the treatment of phantom limb pain [7–9] and has also begun to show promise as a therapeutic approach for stroke [5]. Research has demonstrated that MT is an effective adjunctive therapy for improving upper extremity motor function and daily living among stroke patients [7, 10–12]. A study found that MT combined with bilateral arm training and graded activities was effective in improving motor performance of the paretic upper limb after stroke compared with conventional therapy without MT [13]. We have also done previous studies demonstrating that the combination of conventional rehabilitation therapy and TBMT is an effective way to improve functional recovery in upper limb stroke patients [14] (Fig. 1). However, to date there has been little agreement on the facilitative effect of MT. A randomized controlled trial by Antoniotti et al. found no significant difference between actual and sham MT for functional recovery of the upper extremity in early stroke [15]. There is a strong link between acquisition of motor skills and neuronal plasticity at cortical and subcortical levels in the central nervous system [16]. Therefore, it is possible that these limitations arise from the fact that patients exhibit varying degrees of structural preservation at the injury site, and the treatment of MT effect on upper limb motor function of hemiplegia is somehow dependent on the severity of the lesion, which ultimately resulted in contradictory findings.

The corticospinal tract (CST) is the primary descending motor pathway that connects cortical motor regions with spinal cord neurons, and it plays a crucial role in human upper limb mobility. Consequently, maintaining the structural integrity of the CST is crucial for determining the upper limits of upper limb recovery in patients. Studies have shown that the extent of CST injury is the optimal biomarker for successful rehabilitation of the upper extremity after stroke [17]. Baseline anatomical integrity assessment is essential to ensure patient balance and uniformity. Furthermore, this approach can predict functional recovery in chronic stroke for more effective neurological rehab treatment. Because there is a significant correlation between functional recovery and the preserved structural basis of the lesion [18]. Diffusion tensor imaging (DTI) is an MRI imaging technique that can detect lesions and display nerve fiber bundles in three dimensions [19]. Previous research has established that extracting CST bundle FA values from DTI data can yield valuable insight into their integrity and provide useful information about individualized rehabilitative strategies in stroke patients [20]. For this study, in an attempt to ensure the consistency of treatment efficacy irrespective of patient disease characteristics, we will employ DTI to assess the severity of CST degeneration in our subject patients. Post-enrollment, we will conduct a subgroup analysis to ensure balanced rehabilitation potential across various patient populations.

On the other hand, imaging evaluation can also help us understand the mechanisms by which mirror therapy [21–23]. For example, during MT, stroke patients showed increased functional magnetic resonance imaging (fMRI) activities across widely distributed brain regions [24, 25]. Camera-based visual input from the start-up mirror facilitated motor recovery, daily functioning, and brain network separation in subacute stroke patients [26]. However, few authors have been able to provide a systematic account of the mechanisms of mirror therapy. This new clinical trial is unique due to the following factors: the innovative utilization of DTI imaging which considers CST integrity as a fundamental measure, reduces variables that may impact the randomization process; the implementation of a novel therapeutic apparatus that improves the effectiveness of TBMT; to investigate the mechanism, the project proposes to use the BOLD-fMRI technique to reflect the active level of brain regions and whole-brain functional network connections to reveal the spontaneous activity patterns, connectivity patterns, and brain network characteristics, thus providing new theoretical support for the study of the neural mechanisms of mirror therapy.

## Methods/design

### Study design

This is a single-center, prospective, single-blinding, randomized controlled superiority trial. The objective will be used to evaluate the effectiveness of TBMT (using a newly designed mirror) compared to conventional occupational therapy on upper extremity functions of stroke patients. Patients will be randomly allocated in a 1:1 ratio into a control (*N* = 25) or TBMT group (*N* = 25), with the control group receiving conventional occupational therapy intervention and TBMT group receiving task-based mirror therapy in addition to CG. A professional team will measure relevant outcomes at baseline and 4 weeks after treatment.

### Sample size estimation

The sample size was derived from pre-existing research using G Power v.3.1, considering significance level (*α*) = 0.05, power (1 − β) = 0.95, and given that the minimal clinically significant difference in patient’s hand function improvement, as per prior study data, is 9 points in the FMA-UE with an effect size of *d* = 1.13 [14]. Estimation via G Power software yielded a requirement of 44 samples. Considering potential subject attrition or termination, the sample size could be augmented by 10–15%, resulting in the adjusted sample size of *N* = 50, or 25 patients in each group. Consequently, the total sample size for both groups was formulated as 50.

### Participants

Fifty eligible patients with upper limb motor dysfunction in stroke will be recruited from the outpatient and inpatient departments of the Department of Rehabilitation Medicine, Department of Neurology, and Department of Neurosurgery of the Second Hospital of Chongqing Medical University. Eligible patients had to satisfy the inclusion, exclusion criteria, and termination criteria (Table 1). Information about this trial will be posted via posters in the waiting area of the Rehabilitation Department and the Rehabilitation Medicine Clinic so that interested patients can contact the program director through their therapist. If an applicant meets the study criteria, then they will be invited to participate

**Table 1:** Inclusion, exclusion criteria and termination criteria

**Inclusion criteria** 1. Meet the diagnostic criteria for stroke (including cerebral hemorrhage and cerebral infarction) formulated by the 4th National Cerebrovascular Disease Conference in 1995and confirmed by head CT or MRI, and the course of the stroke is 1 to 6 months 2. Abnormal hand function after stroke, Brunnstrom stage I-III, upper limb FMA-UE score lower than 47 3. The contralateral hand is in good condition 4. The age of the patient is 20-80 years old, and the gender is not limited 5. No progressive stroke performance, stable vital signs 6. Cognitive function is relatively intact, MMSE>17 or MoCA>24 7. Be able to follow the clinical treatment plan and sign the subject's informed consent
**Exclusion criteria** 1. Subjects with upper limb dysfunction before stroke 2. Subjects with visual impairment or unilateral neglect 3. Severe cognitive impairment or poststroke depression 4. Metal implants in the body, such as cardiac pacemakers and other metal devices 5. Suffering from serious underlying diseases, such as heart function level 4 (NYHA) or severe COPD 6. Subjects participating in other clinical trials at the same time 7. Subjects judged to be unsuitable for this trial, and withdrawing voluntarily 8. Subjects unable to undergo MRI scans
**Termination criteria** 1. Subjects have serious adverse events or adverse reactions 2. Subjects have other changes in their condition and cannot continue the trial 3. Subjects have significant problems in the design of the protocol or the trial and are unable to determine the efficacy

### Randomization and blinding

A total of 50 patients who meet the inclusion criteria will be randomly assigned to either the task-based mirror group or the control group. The randomization sequence will be generated by an independent statistician using SPSS Version 19.0. Patients will be assigned randomization numbers based on the order of enrollment. To maintain blinding, opaque sealed envelopes will be utilized to conceal group assignments. Patients will only become aware of their group after opening the envelope, which will be securely stored in a separate cabinet. Blinding of evaluators will be employed in this study to avoid bias stemming from subjective factors. The same investigator, who will be blinded to the treatment assignment, performed all the assessments. Additionally, statisticians will conceal the groupings and their significance, and the code will remain undisclosed until the analysis is finalized. In case of a severe incident during a trial, blinding may need to be undone for proper treatment. This decision must be endorsed by the trial’s lead researcher/medic, and recorded in the protocol, with utmost consideration given to minimizing bias and ensuring data quality.

### Interventions

**Fig. 2:**
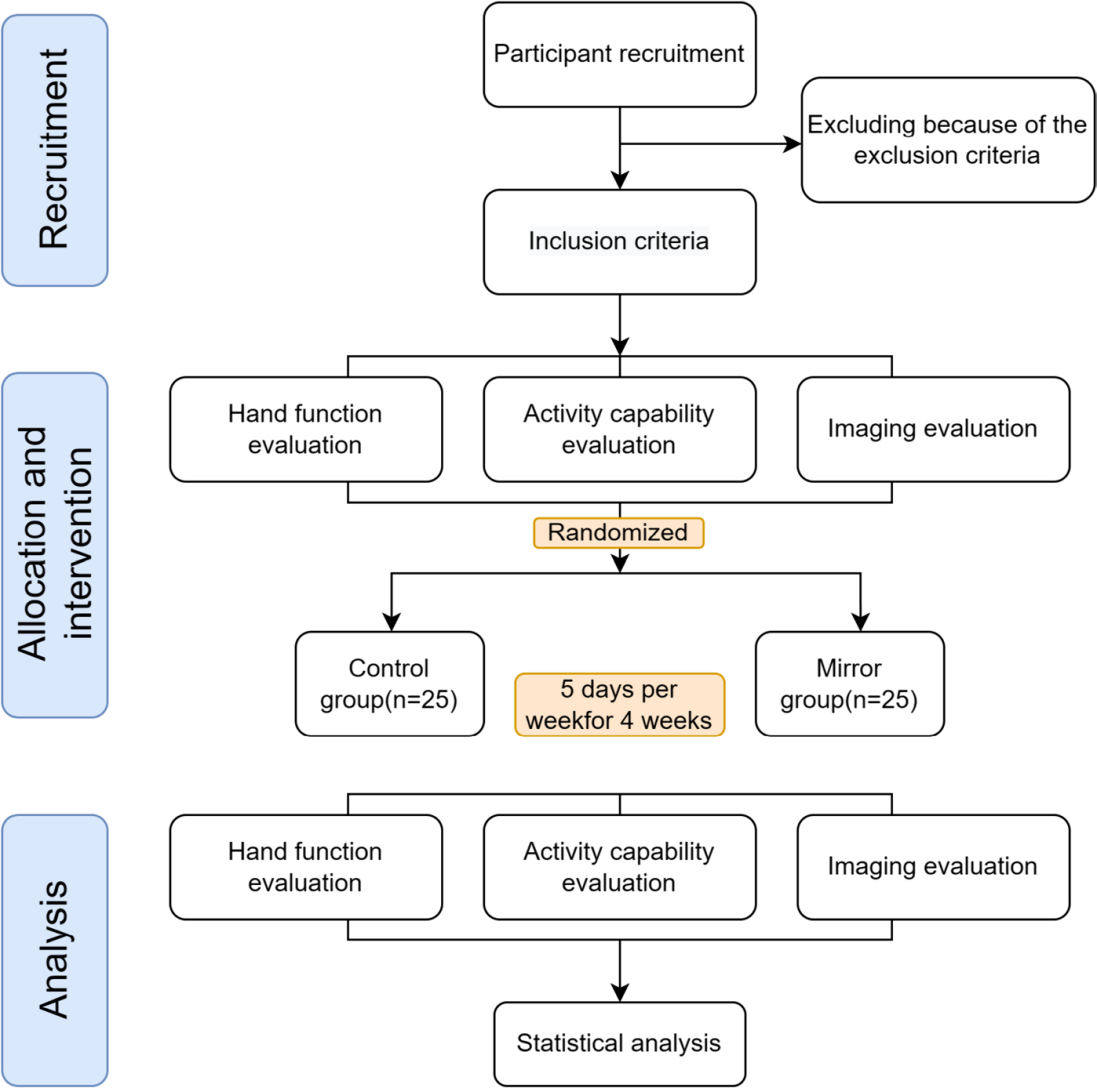
Proposed participant flow

The study protocol is illustrated in Fig. 2. All participants who fulfil the inclusion criteria will be assessed. This research protocol conforms to the CONTORT 2010 guidance, adheres to the Revised Standards for Reporting Intervention trials (SPIRIT) protocols, and satisfies the SPIRIT checklist criteria (online supplementary additional file 2) [27]. This trial will investigate the TBMT versus a control intervention and accords with the SPIRIT Figure (Table 2). Patients will be randomized to a conventional and task-based mirror treatment group for 5 consecutive days per week for 4 weeks, ensuring a total of 40 min of treatment time per day for each group. A trained and professional occupational therapist will be assigned to conduct this trial, and each patient will sign an informed consent form (online supplementary additional file 1) before treatment.

**Table 2:**
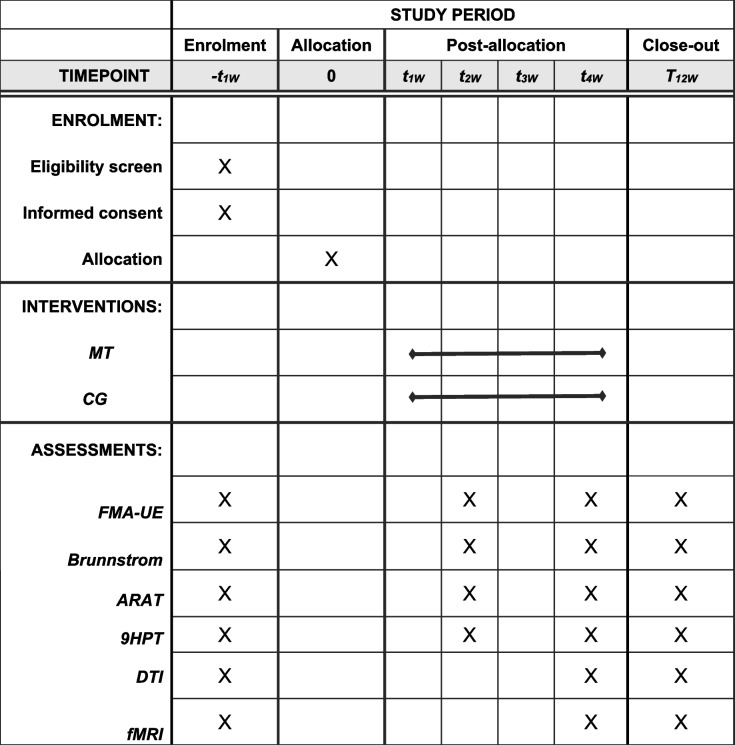
Schedule of enrollment, intervention, and assessment

### Control group (CG)

Depending on the patient’s condition, 20 min of occupational therapy without the mirror will be conducted alongside 20 min of conventional treatment, resulting in a total duration of 40 min. The above training will be performed unassisted by a mirror therapy rehabilitator 5 days a week for 4 weeks.

### Task-based mirror therapy (TBMT)

**Fig. 3:**
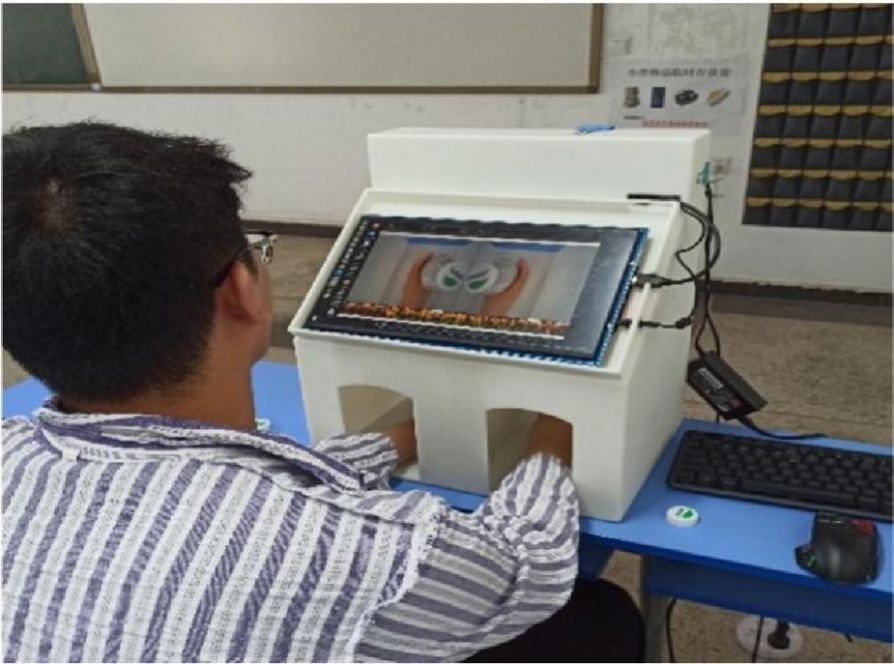
Ergonomic mirror therapy device

Patients in the TBMT group will receive 20 min of conventional therapy followed by 20 min of TBMT. We designed an ergonomic mirror therapy device (patent number: Z120212 2278905.3) (Fig. 3). Patients will be seated facing the mirror device, with both hands in the box under the screen that substitutes the conventional mirror. The high-definition LCD can display the mirrored image of the healthy hand. Both hands can move freely in the box. By looking at the image on the LCD screen, the patient may create an “optical illusion” that activates mirror neurons and triggers the active activity of the affected hand. The task orientation is as follows: the patient moves with the healthy limb while observing the mirror image and using the affected hand as much as possible to perform the following movements together: elbow flexion and extension, ulnar and radial deviation, wrist flexion and extension, finger flexion and extension, finger adduction, and abduction, as well as the use of sponge balls, water cups, rags, chopsticks, cups, tubes, and wooden blocks. Patients will use their healthy upper limbs to move objects such as watches and rings to promote imagination. This process focuses on placing an object on the affected limb while exercising on the healthy side to facilitate stimulation. The affected upper limb may not have any allowed voluntary activity [14].

Appropriate care is permitted during the trial period, including pain management, defecation assistance, and posture management. Hand function interventions are prohibited during non-treatment periods to ensure the effectiveness of the trial.

### Outcome measurements

Patients will undergo clinical outcome measurements at baseline, 4 weeks into treatment, and immediately after 12 weeks of treatment. Evaluations will encompass basic information, indicators of hand function, daily activity, and imaging techniques (DTI, CST integrity assessment, including FA, rFA, ROI, and FC values). Specialized training will be provided to evaluators to ensure the credibility of the evaluation.

#### Primary outcomes

The Fugl-Meyer Assessment for upper extremity (FMA-UE) will be utilized to evaluate the level of upper limb motor function impairment, with a maximum score of 66 points [28, 29]. This assessment includes 23 different movements grouped into four categories (shoulder/elbow/forearm, wrist, hand/finger, and coordination), whereby 33 items are assessed. Rated on a 3-point scale, task completion is scored as 0 for inability, 1 for partial ability, and 2 for near-normal ability. The reliability, validity, and responsiveness of the FMA-UE assessment for stroke patients are well recognized [30]. Therefore, FMA-UE will be considered as the primary outcome.

#### Secondary outcomes

Brunnstrom is a motor function and muscle spasticity assessment method for the affected upper extremity [31]. It includes six stages: Grade 1: absence of random movement, no upper or lower extremity movement; Grade 2: joint response, comovement, and minimal random movements; Grade 3: random comovement with the ability to grasp but not extend; Grade 4: separation of movement, including the ability to pinch and make minor extensions; Grade 5: gradual restoration of muscle tone with fine movements, simultaneous yet independent extension of fingers; and Grade 6: motor ability approaching normalcy but speed and accuracy are suboptimal.

The Modified Barthel Index (MBI) assesses the mobility of stroke patients concerning their ability to carry out specific activities of daily living. These activities include eating, bathing, dressing, bowel and urinary control, toileting, bed and chair transfer, level walking, and stair walking [32, 33]. A total score of 100 can be achieved, and scores of 60 or higher indicate self-sufficiency. Independence positively correlates with the score.

We commonly use the Action Research Arm Test (ARAT) to assess the ability of the affected upper extremity and hand to handle objects of different sizes, weights, and shapes. The test assesses 19 tests of arm motor function, both distal and proximal. Each test is given a score of 0, 1, 2, or 3, with higher scores resulting in better function. The total ARAT score is the sum of the 19 tests, resulting in a maximum score of 57 [34]. Nine Hole Peg Test (9HPT): assesses the patient’s whole hand dexterity. The assessment requires the patient to take nine pegs from the container and insert them into a small pegboard, then remove and place them back into the container. The patient’s score depends on the speed of insertion and removal, with the faster the time, the higher the score [35].

### Imaging outcome measures

**Fig. 4:**
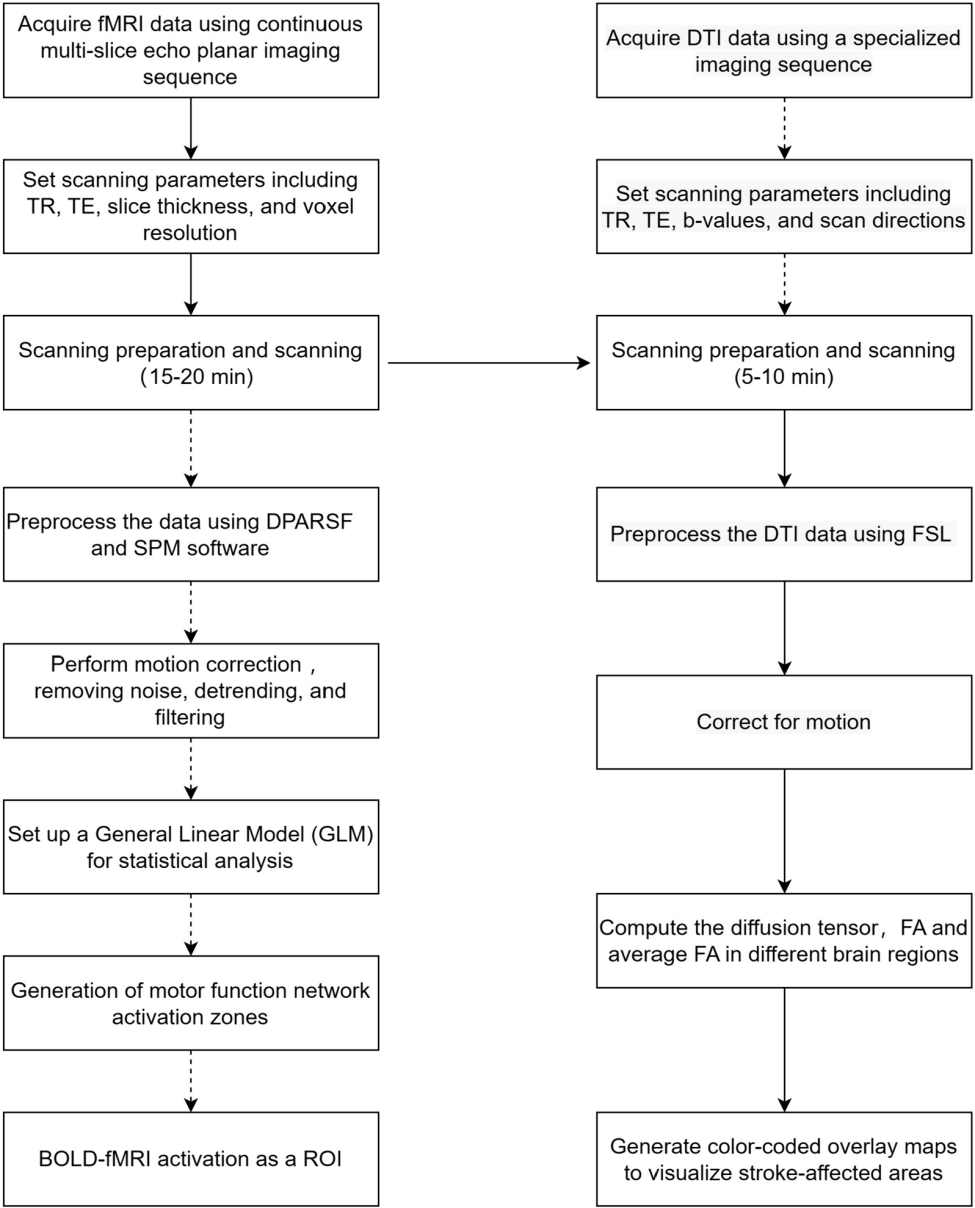
The DTI and fMRI scanning workflow diagram

Subjects will undergo Rs-fMRI scans utilizing a gradient echo EPI sequence (repetition time = 2000 ms, echo time = 30 ms, FOV = 220 × 220mm^2^, flip angle = 90°, matrix size = 64 × 64, 30 transverse slices, slice thickness = 4 mm, gap = 0.8 mm). Each Rs-fMRI acquisition will yield 240 volumes [36]. High-resolution T1-weighted 3D anatomical images will be obtained in the sagittal orientation using a fast field echo sequence, repetition time (TR) = 7.4 ms; echo time (TE) = 3.6 ms; flip angle = 8^◦^; field of view (FOV) = 250 × 250 mm^2^; matrix = 228 × 227; 150 slices; slice thickness = 1.1 mm with no gap and voxel size = 1.1 × 1.1 × 1.1 mm^3^. DTI parameters were a spin echobased echo-planar imaging sequence in contiguous axial planes that included 16 volumes with diffusion gradients applied along 15 noncollinear directions (*b* = 800 s/mm^2^) and one volume without diffusion weighting (*b* = 0 s/mm^2^). Each volume consisted of 65 contiguous axial slices covering the entire brain (TR = 6973 ms; TE = 75 ms; flip angle = 90^◦^; FOV = 224 × 224 mm^2^; matrix = 112 × 112 and voxel size = 2 × 2 × 2 mm^3^). All MRI scans need to be performed by a specialized imaging physician, with the patient being scanned for both fMRI and DTI sequences in a single session, with the fMRI scan taking approximately 15–20 min, followed by the DTI scan taking approximately 5–10 min. The DTI and fMRI scanning workflow diagram is shown in Fig. 4.

### Image data acquisition and preprocessing

For fiber tract identification, we will use automated fiber quantification (AFQ) software to identify 20 WM tracts in each participant’s brain [37]. The identification procedure included three primary steps: (1) whole-brain deterministic fiber tractography, (2) waypoint ROl-based tract segmentation, and (3) probability map-based fiber refinement. Twenty fiber tracts will be identified according to the predefined ROls and probability maps: bilateral thalamic radiation CST, cingulum cingulate, cingulum hippocampus callosum forceps major, callosum forceps minor, bilateral inferior front-occipital fasciculus (IFOF), bilateral inferior longitudinal fasciculus(lLF), superior longitudinal fasciculus (SLF), bilateral uncinate, bilateral arcuate. Then, each fiber bundle will be divided into 100 segments. Finally, the FA, MD, AD, and RD of each segment of each fiber bundle will be calculated. The automated calculation will be performed by MATLAB 2011b. First, the effective self-diffusion tensor will be computed from the movement and distortion-corrected dataset [38]. The unit-less FA is an anisotropy index and ranges from 0 in fully isotropic diffusion to 1 in fully anisotropic diffusion [39]. Mean FA will be calculated for the affected and unaffected sides of each patient within a 5-mm radius of the posterior limb of the internal capsule. For methods, see Radlinska et al. [40]. Based on this, the diffusion indicators of rFA and FAasy are calculated for both participant groups using the following formula:

$$\begin{array}{l}rFA=\frac{F{A}_{ipsilesional}}{F{A}_{contralesional}}\\ F{A}_{asy}=\frac{F{A}_{contralesional}-F{A}_{ipsilesional}}{F{A}_{contralesional}+F{A}_{ipsilesional}}\end{array}$$

The values of rFA and FAasy ranged from 0 to 1. The lower the rFA and the higher the FAasy, the more significant the decrease in ipsilateral CST anisotropy of the lesion, i.e., the more severe the impairment of CST integrity.

### fMRI

The fMRI is an imaging technology without intervention and employs magnetic resonance for quantifying hemodynamic alterations triggered by neuronal activity, indicating brain region activation. On patient readiness, full brain scans commence during rest-state scanning, instructing them to unwind, occlude vision, and refrain from intentional cognitive tasks. Images of the patients with left‐sided lesions will be flipped before preprocessing. After flipping, the right hemisphere will be tagged as the ipsilesional side and the left hemisphere as the contralesional side. fMRI preprocessing will be carried out using DPARSF, which provides a pipeline workspace based on Statistical Parametric Mapping (SPM; Wellcome Trust Centre for Neuroimaging, London) [41]. First, volumes from each run will be realigned to their first volume to estimate motion artifacts. The head motion will be then corrected using a six‐parameter rigid body spatial transformation. Functional mean images will be obtained and co‐registered to the corresponding individual structural image before normalization to the standard Montreal Neurological Institute (MNI) template. Finally, images will be smoothed with an 8‐mm isotropic Gaussian kernel.

### Functional connectivity analysis

In this study, seed-based analysis will estimate brain region connectivity strengths by calculating correlation coefficients of each brain area’s time series. ROIs will be identified from brain areas differing between healthy and stroke patients with impaired hand function. Pearson correlations between ROIs and all brain voxel time series will be assessed, transforming them into normal distributions through Fisher’s *z* transformation. A whole-brain functional connectivity analysis, seeding on the abnormal brain area, will identify the post-mirror therapy functional connectivity differences between the control and experimental groups [36].

### Whole-brain functional network analysis

Following preliminary processing, the representative time series of each region of interest (ROI) will be acquired by extracting and averaging the time series of all the voxels within the specified ROI. An asymmetric correlation matrix will be generated by computing Pearson’s correlation coefficient of all conceivable pairs of the representative time series. Sparsity (Sp) will be used to set the threshold value and calculate the brain network topology properties. Finally, the differences in the topological properties and connections of brain networks between the intervention and control groups will be explored by analyzing the whole-brain functional network.

Experiments consider conducted employing the SPM8-compliant DPARSFV2.2 suite on MATLAB 2011b. Paired *t*-tests will analyze data sets from individual groups before and after intervention, with *p* < 0.05 defining significance. Later, a two-sample *t*-test will assess differences in functional connectivity between the study and control cohorts post-therapy. A *p* < 0.05 cut-off will be utilized for substantial functional connectivity alterations, followed by AlphaSim multiple comparison corrections. Relevant observation zones consist of activated areas with ≥ 82 active voxels.

### Safety and adverse event monitoring

There will be a low probability of adverse events in this study. Potential risks are skin irritation, redness, scratching, dizziness, and seizures. Any adverse events during the study should be recorded, observed, and followed up in detail, including whether the adverse event is quantitatively related to mirror therapy, whether symptoms resolve or disappear after stopping treatment, and whether appropriate treatment has been administered. The questionnaires will be administered to the participants after every treatment, and the results will be recorded and analyzed. The outcome assessors will judge the severity (mild, moderate, or severe), seriousness, and causality (definitely related, probably related, possibly related, possibly not related, definitely not related to the intervention, or not assessable). In severe cases, more moderate interventions are required to prevent overwhelming the patient’s body. In the event of severe adverse reactions or worsening of the condition, immediate termination of the experiment and prompt reporting to the ethics committee are essential. In addition, a Data Monitoring Committee (DMC) will help finalize interim analyses or stop rules. The DMC is composed of clinicians and statisticians, independent of the sponsor and study team. It scrutinizes accumulated data and recommends modifications for future trials, ensuring risks to participants remain within limits. The DMC can discontinue a trial if significant protocol infractions transpire or large patient dropouts occur. Should any participants suffer any harm from the trial, they will receive necessary medical treatment for the injury or complication, free of charge, as a public patient within the Second Affiliated Hospital of Chongqing Medical University. This safeguard aims to protect the health and safety of participants and provide necessary medical care.

### Statistical analysis

Data management and analysis will be conducted in SPSS version 23. Groups will be compared at baseline using the *t* test for independent samples for the continuous variables, and the chi-square test for categorical data. The differences of the outcomes, including the FMA-UE, MBI, ARAT, and the 9HPT, between the groups at each of the trial time points will be analyzed using mixed-design 2-way repeated-measures analysis of variance (ANOVA), taking time (4 levels: preintervention, after 2 weeks, after 4 weeks of intervention and 3 months at the end of the trial for follow-up) as the within-subject factor and group (2 levels: TBMT and CG) as the between-subject factor. If participants drop out during the intervention period, they will not be excluded due to the principle of intention to treat (ITT). Furthermore, in instances of missing data, the continuation of observations (LOCF) method will be employed, whereby the last recorded observation preceding the gap in data will serve as a substitute. Unfavorable stroke outcomes correspond to lower FA values for corticospinal tracts, while robust functional recovery denotes elevated FA values. Subgroup analyses of FA values will be conducted based on the interquartile spacing (< 25% interval; 25–75% interval; > 75% interval) method post the inclusion of all individuals to standardize the baseline conditions of the patients. We will use linear regression model outcomes at 4 weeks post-treatment cessation as the dependent variable (FMA-UE). This model will include treatment assignment (TBMT vs. CT) and baseline outcome measure as fixed-effects, as well as patient characteristics (low or high impairment severity at baseline). We will examine differential impacts by fitting separate models with treatment-impairment severity. We will report relevant point estimates and standard errors for these models and conduct 5% level tests for interaction terms with two-sided alternative hypotheses. A *p*-value < 0.05 is deemed a statistically significant discrepancy.

### Patient and public involvement

Although patients cannot directly participate in the design process of clinical trials, they can offer valuable input during the recruitment and informed consent phase. Their views can enhance transparency and fairness, ensuring that patients fully understand the purpose, risks, and advantages of the trial. We prioritize regular feedback and evaluation from patients and assess their satisfaction with the trial process each time to ensure that their voices are heard and to identify necessary improvements. The outcome measures to be evaluated in this study will potentially be influenced by the sociodemographic characteristics and preferences of the patients. Therefore, we use a comprehensive assessment. The result of our study will be published in a peer-reviewed journal.

### Modification of the protocol

Any modifications to the protocol, including changes in objectives, design, patient population, sample size, and study procedures will require a formal application from the Institutional Review Board (the Board) and Hospital Research Ethics Committee (the Committee) of the Second Affiliated Hospital of Chongqing Medical University. Based on safety assessment data, if research is changed to eliminate an apparent immediate danger(s) to the subject, the investigator will promptly notify the Board and the Committee on Change(s). Review by the Board and Committee at the next meeting convened to determine whether the proposed change(s) are consistent with the subject’s continued welfare.

### Data management and quality

Only researchers who participated in this study can access the data. Each participant will receive an ID number for privacy during the trial period. Details are captured on custom case report forms (CRFs) that undergo double checking for accuracy and completion. Associated documents obtain unique code numbers then securely stored within the Rehabilitation Medicine Division of the hospital. Hard copies are transferred to an Excel spreadsheet for review by two doctors before storage on a password-protected computer. Only the lead researcher and statistician hold passwords to this document cabinet, adhering to medical record retention laws and GCP principles. Two additional healthcare professionals will undertake periodic physical site audits to confirm meticulous adherence to the investigative protocol and data accuracy. Through these strategies, we augment patient cooperation and decrease instances of dropout.

## Discussion

Hand function is a key issue in stroke rehabilitation. While there are various treatment options, the availability of precise and effective methods remains a major challenge. Mirror therapy is a relatively effective therapeutic intervention [14]. However, previous studies have not linked functional recovery to the structural basis preserved by the lesion. This may be a potential reason why different teams in the current mirror therapy study have reached opposite results [42]. Therefore, it is imperative to establish high-quality evidence to support the use of mirror therapy in stroke rehabilitation. To address this gap, this is the first study to incorporate comprehensive patient baseline data while considering structural preservation and utilized a customized ergonomic therapeutic device. The primary aim of this study is to assess how effective MT is in rehabilitating stroke patients.

MT is based on the mirror neuron system and uses the plane mirror imaging principle for the contralateral activity images projected on the affected side. It also combines optical illusions, visual feedback, and virtual reality [43]. This visual stimulus supports motor rehabilitation by capitalizing on the strong connection between visual input and the pre-motor cortex [44]. A recent clinical study concluded the neural mechanisms of motor imagery training (MIT) in stroke rehabilitation by fMRI. The conclusion was drawn that MIT helps reduce compensatory activation in both hemispheres in stroke patients, remodels FC in the ipsilateral hemisphere, and promotes functional recovery [45]. However, there is a relatively small body of literature that is concerned with the neural mechanisms of mirror therapy in depth. In this study, we will focus on analyzing the Pearson correlation coefficients between two specific brain regions. We will also investigate the differences in topological properties and lateral connections within the brain network of both intervention and control groups. By doing so, we aim to identify any characteristic manifestations of brain network changes resulting from mirror treatment. This will enable us to better understand the neurological mechanism behind improved hand function through mirror treatment. Meanwhile, we will use an array of assessment techniques to determine the effectiveness of TBMT, including autonomic control (FMA), arm and hand motor recovery (Brunnstrom phase), manual dexterity (9HPT), and daily activities. This study is extremely comprehensive, as you can observe.

In conclusion, this will be a well-controlled, high-quality clinical study, and the findings may be used to update evidence-based protocols in postacute stroke care planning and to translate evidence into practice and medical decision-making.

### Supplementary Information

**Supplementary Material 1.**

**Supplementary Material 2.**

## Data Availability

Not applicable.

## Abbreviations

DTI: Diffusion tensor imaging
FMA-UE: Fugl-Meyer Assessment Upper Extremity Scale
MBI: Modified Barthel Index
ARAT: Action Research Arm Test
9HPT: Nine Hole Peg Test
TBMT: Task-based mirror therapy
MT: Mirror therapy
CST: Corticospinal tract
fMRI: Functional magnetic resonance imaging
BOLD-fMRI: Blood oxygenated level dependent functional magnetic resonance imaging
OT: Occupational therapy
FA: Fractional anisotropy
rFA: Relative fractional anisotropy
ROI: Region of interest
FC: Functional connectivity
EPI: Echo-planar imaging
DPARSF: Data Processing Assistant for Resting-State fMRI
SPM: Statistical Parametric Mapping
MNI: Montreal Neurological Institute
GCP: Good Clinical Practice
MIT: Motor imagery training
