# Supplementary material for: Effects of task-based mirror therapy on upper limb motor function in hemiplegia: study protocol for a randomized controlled clinical trial
Source: Trials. 2024 Apr 11;25:254. doi: 10.1186/s13063-024-08081-1 (PMC11010366; doi:10.1186/s13063-024-08081-1)
Supplement: Supplementary file 1 — Supplementary Material 1. [file 13063_2024_8081_MOESM1_ESM.docx]

INFORMED CONSENT FORM

Notice to respondents

**Dear Prospective Participant**

You are invited to participate in the research project "Task-based mirror therapy enhances upper limb motor function in stroke patients". This study was reviewed and approved by the Research Ethics Committee of Chongqing Medical University. Your participation in this study is voluntary, and this information can help you decide whether or not to participate in this scientific study. Please read it carefully and ask the study coordinator any questions you may have.

1. Purpose and background of the project

Upper limb motor dysfunction is one of the most common functional impairments after stroke, which seriously affects patients' daily activities and delays functional recovery. We aim to investigate the effectiveness of task-based mirror therapy on hand motor function after stroke compared to mirror therapy alone and to provide a basis for the rehabilitative effect of this treatment on upper extremity motor function in stroke patients.

1. Explanation of Procedures

If you volunteer for the study, we will communicate with you or your family in detail to inform you about the study. After participating in this study, we will record your basic information, your baseline medical condition, and the results of some tests you have had. After collecting this basic information, we will also perform other program-related assessments, such as muscle strength and tone measurements and cognitive status assessments, for you free of charge. The assessment will be completed at the hospital or by telephone follow-up. The content and steps of the test will be explained by the researcher. In addition, we will number each participant and create a research profile.

1. Discomfort and Risks

For you, follow-up visits will require telephone contact with you or your agent and will require you and your agent to cooperate in some rehabilitation evaluations, which may be troublesome or inconvenient for you.

1. Benefits

All assessments related to your participation in the research process will be free of charge, and you can obtain professional evaluation and rehabilitation advice to help you learn more about your disease and motor function status. In addition, the study of your information will facilitate the acquisition of scientific data, which will provide significant evidence for disease diagnosis and treatment, biomedical science research, etc., and generate an important social value.

1. Privacy

If you decide to participate in this research, all information you supply during the research will be held in confidence, and unless you specifically indicate your consent, your name will not appear in any report or publication of the research. Your data will be safely stored in a locked facility, and only research staff will have access to this information. The responsible investigator, other investigators, and members of government regulatory authorities or ethics review committees have access to or use of your medical records as needed. This information may include your name, address, telephone number, medical history and information obtained in connection with your evaluation.

1. Voluntary Participation

Your participation in the study is completely voluntary, and you may choose to stop participating at any time. Your data will not be included in the study results and any of your rights will not be affected as a result.

You can keep yourself informed about the information and progress of the study, and if you have any questions related to the study, if any discomfort or injury occurs during the study, or if you have questions about the rights of the

participants in the study, you can contact the study leader: Botao Tan (tel. 18723866166).

Subject Statement:

I have been informed about the purpose, background, process, risks and benefits of this study.

I have enough time and opportunity to ask questions, and I have had all my questions answered to my satisfaction.

I was also told who to contact when I had questions, wanted to reflect difficulties, concerns, suggestions for research, or wanted further information or help with research.

I have read and understand the explanation provided to me, and I voluntarily agree to participate in this study.

I have been informed that I can choose not to participate in this study or withdraw at any time with notice to the investigator without discrimination or retaliation and that any of my rights will not be affected as a result.

The investigator may terminate my continued participation in this study if I do not comply with the study plan, if a study-related injury occurs or for any other reason.

I will receive a signed copy of the "Informed Consent Form" that includes my signature and that of the investigator.

Signature of Participant: Phone of Participant: Date:

I have accurately informed the subject of this document, and he/she has accurately read this form and had the opportunity to ask questions.

Signature of investigator: Phone of Participant: Date:

(Note: The signature of a witness is required if the subject is illiterate, and the signature of a proxy is required if the subject is incapacitated)
